# Recent advances in selective photothermal therapy of tumor

**DOI:** 10.1186/s12951-021-01080-3

**Published:** 2021-10-24

**Authors:** Liping Zhao, Xu Zhang, Xiaoxia Wang, Xiuwen Guan, Weifeng Zhang, Jinlong Ma

**Affiliations:** 1 College of Pharmacy, Weifang Medical University, Weifang, 261053 China; 2 School of Clinical Medicine, Weifang Medical University, Weifang, 261053 Shandong China; 3 Collaborative Innovation Center for Target Drug Delivery System, Weifang Medical University, Weifang, 261053 Shandong China; 4 Shandong Engineering Research Center for Smart Materials and Regenerative Medicine, Weifang Medical University, Weifang, 261053 China

**Keywords:** Photothermal therapy, Selective killing, Targeted enrichment, Self-regulating

## Abstract

Photothermal therapy (PTT), which converts light energy to heat energy, has become a new research hotspot in cancer treatment. Although researchers have investigated various ways to improve the efficiency of tumor heat ablation to treat cancer, PTT may cause severe damage to normal tissue due to the systemic distribution of photothermal agents (PTAs) in the body and inaccurate laser exposure during treatment. To further improve the survival rate of cancer patients and reduce possible side effects on other parts of the body, it is still necessary to explore PTAs with high selectivity and precise treatment. In this review, we summarized strategies to improve the treatment selectivity of PTT, such as increasing the accumulation of PTAs at tumor sites and endowing PTAs with a self-regulating photothermal conversion function. The views and challenges of selective PTT were discussed, especially the prospects and challenges of their clinical applications.

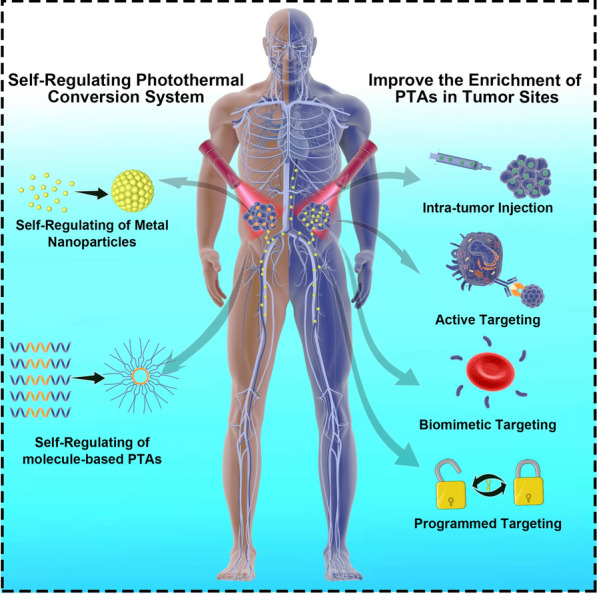

## Introduction

Cancer therapy is one of the most significant challenges facing the health care industry today [1]. According to a recent survey, in 2020, the number of new cancer patients globally is approximately 19.29 million, and the number of deaths has reached 9.6 million. Cancer’s high incidence and mortality have led researchers worldwide to work hard to develop more accurate and rapid diagnostic strategies and effective anticancer methods [2, 3]. As an effective treatment, traditional treatments (chemotherapy, radiotherapy, and surgery) are the most commonly employed clinical treatment methods. However, patients may have a high risk of treatment failure or posttreatment side effects during or after traditional treatment [4, 5]. Among the emerging cancer therapies, photothermal therapy (PTT) utilizes the photothermal effect of photothermal agents (PTAs), which converts absorbed light energy to heat to cause thermal burns on the tumor. PTT has high research value because of its simple operation, short treatment time, and rapid recovery [6, 7]. More importantly, PTT is a highly effective and noninvasive therapy that can eliminate various types of cancer. It is well known that the ultimate goal of cancer treatment is to kill cancer cells without damaging normal cells [8–10]. The greatest problem of PTT is the systemic distribution of PTAs in the body and non-precision exposure of lasers, which can cause serious side effects on normal tissues around tumors when using existing PTAs for PTT [1].

**Scheme 1:**
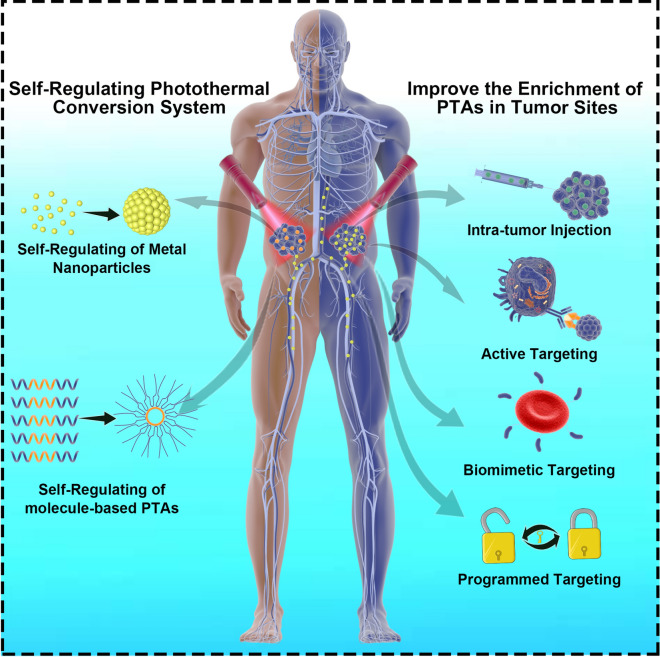
Schematic of strategies for improving selective photothermal therapy

Increasingly, researchers have recognized the advantages of selective killing of tumor cells in PTT and developed a variety of strategies to achieve selective killing by PTT (Scheme 1), such as increasing the concentration of PTAs at the tumor site and giving PTAs a self-regulating photothermal conversion capability [1, 11–14]. The simplest and most universal solution is to increase the enrichment amount of PTAs at the tumor site. The concentration difference between normal tissue and tumor tissue can be generated, and the temperature of the tumor site can be selectively increased [15]. The ideal solution is to give PTAs a self-regulating photothermal conversion capability, which means that PTAs have a weak photothermal conversion ability in normal tissue but a strong photothermal conversion ability at the tumor site [13, 14, 16]. Theoretically, the temperature of the tumor site can be selectively increased, with minimal or no damage to normal cells. In this review, we summarized strategies for improving the selective efficiency of PTT and discussed the views and challenges of PTT in the fight against cancer.

## Improve the enrichment of photothermal agents at tumor sites

The process of PTT is the delivery of PTAs to tumor tissue, which is then radiated to raise the local temperature [13, 16]. Therefore, the most straightforward strategy is to increase the concentration of PTAs at the tumor site so that normal tissue and tumor tissue produce a concentration difference, which selectively raises the tumor site temperature [13]. Presently, the most convenient and commonly employed solution is intratumor injection. A large amount of PTAs can be enriched in the tumor tissue, and the temperature of the tumor site can be selectively increased [17, 18]. However, this method is not directly applied for metastatic and deep tissue tumors in the body compared to intravenous injections [19]. To achieve a high abundance of intravenous drugs at tumor sites, nanodelivery systems have been developed from nanoparticles to targeted nanoparticles, biomimetic targeting systems, and programmed targeting systems. These strategies have made significant progress in enhancing the stability of drug circulation and tumor cell uptake [20].

### Intratumor injection

**Fig. 1:**
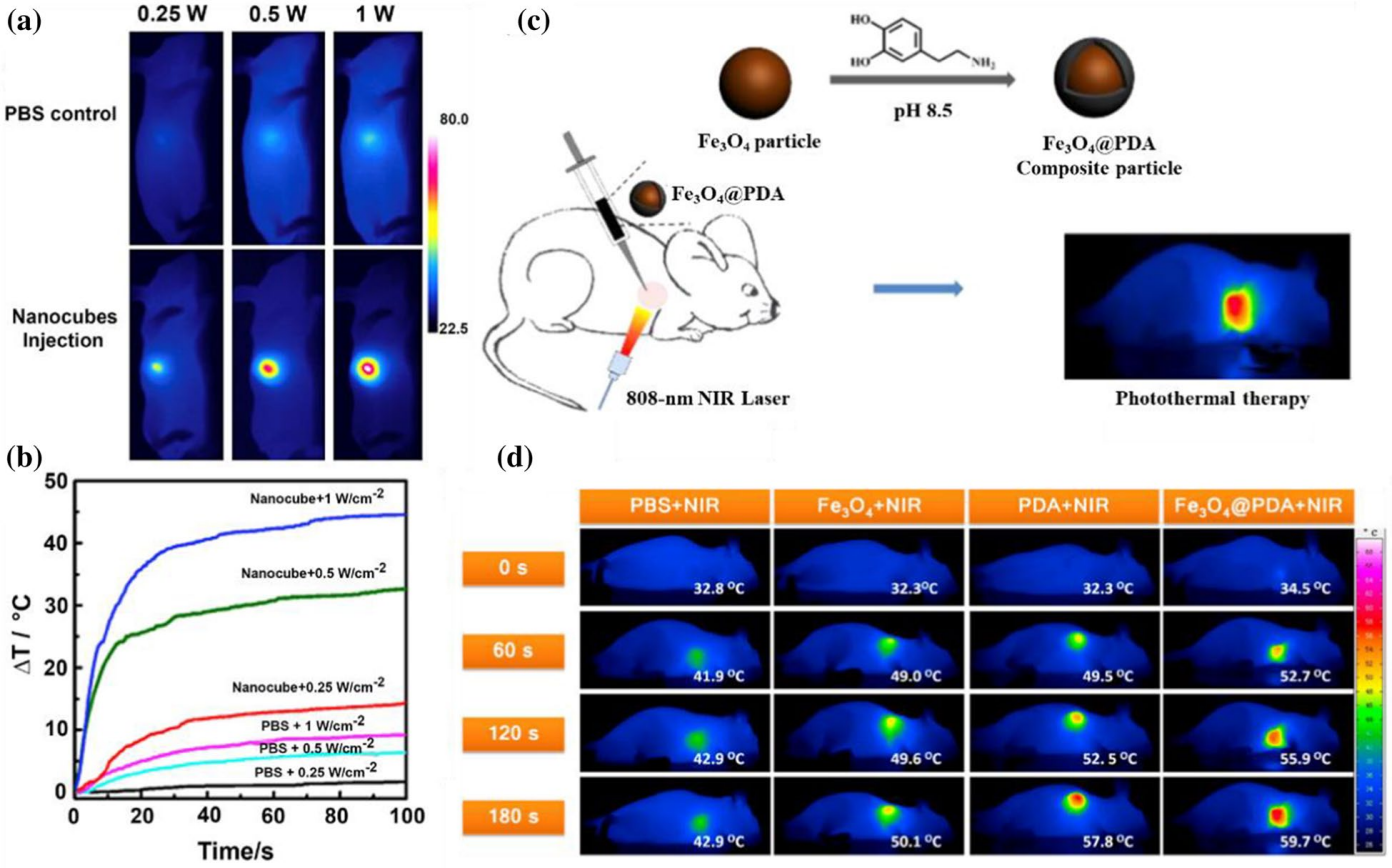
**a** Infrared thermal images and **b** temperature rise curves of 4T1 tumors injected with PBS or (NH_4_)_X_WO_3_ Nanocubes (100 μL of 5.0 mg/mL) after 100 s of 1064 nm laser irradiation (Reprinted from Ref. [23] with permission. Copyright 2015, Elsevier Ltd.). **c** Schematic diagram of intra-tumoral injection of polydopamine coated magnetic composite particles into mice to enhance the photothermal therapy; **d** The infrared thermal image of Fe_3_O_4_, PDA, and Fe_3_O_4_@PDA under NIR laser irradiation (λ = 808 nm; 6.6 W/cm^2^) (Reprinted from Ref. [24] with permission. Copyright 2015, American Chemical Society)

According to a previous study, the amount of PTAs that can reach the tumor site for cancer treatment is much smaller than the amount of intravenous injection due to the devouring effect of the RES system after administration of the whole body and does not have a satisfactory role in the efficacy of the drug [21]. The most striking quality of intratumor injection is its effectiveness in regard to avoiding PTAs loss, which is more conducive to heat ablation of the tumor. Given that light absorbed in the near-infrared second window with a range of 1000–1400 nm has excellent potential to penetrate deep tissue [22]. Haijun Yu et al. prepared (NH_4_)_x_WO_3_ nanocubes, which indicated through in vivo and in vitro studies that (NH_4_)_x_WO_3_ nanocubes have an excellent ability to suppress breast cancer under the second near-infrared window (Fig. 1a, b). After injecting nanocubes and irradiating with a 1064 nm laser in the NIR-II window, they eliminated tumors and inhibited lung metastasis of tumors in mouse models [23]. Polydopamine (PDA) as a mimic of the adhesive proteins found in mussels, shows excellent biocompatibility and biodegradability and has been recently utilized as an effective PTA agent in PTT research. The polydopamine coated Fe_3_O_4_ magnetic composite particles prepared by Shen Shun et al. have a better effect of avoiding interference from the endothelial reticulum system (Fig. 1c, d) [24]. Fe_3_O_4_@PDA particles were injected into the tumors of tumor-bearing mice and then irradiated with a laser. The temperature of the tumor surface rapidly increased to 59.7°C, which demonstrated its excellent photothermal conversion capability.

Via intratumoral injection, nanoparticles enter the tumor. These nanoparticles usually remain at the injection site and have poor permeability in the tumor, leading to incomplete ablation and recurrence [25]. Thus, cell-mediated delivery has great potential in cancer therapy. Nanoparticles can cross nearly impermeable biological barriers to reach target sites that are generally inaccessible to common drugs or nanoparticles [26, 27]. Xue-Feng Yu and colleagues constructed a cell-mediated delivery system using macrophage vehicles to transport BSA-coated Au nanorods (sAuNRs) [28]. Due to their small size, BSA-coated sAuNRs carried by macrophage vehicles exhibited superior anti-phagocytosis because of the better biocompatibility of BSA. After intratumoral injection, macrophages transported, BSA-coated sAuNRs showed greater photothermal conversion efficiency in tumors, and the tumor recurrence rate was the lowest compared with free BSA-coated sAuNRs. However, intratumor injection has some limitations. PTT through intratumor injections can easily damage the external tissue of the tumor and has the risk of spreading cancer cells to other parts of the body. In addition, intratumor injections cannot be used for metastatic tumors and deep tumors [29].

### Tumor targeted enrichment

**Fig. 2:**
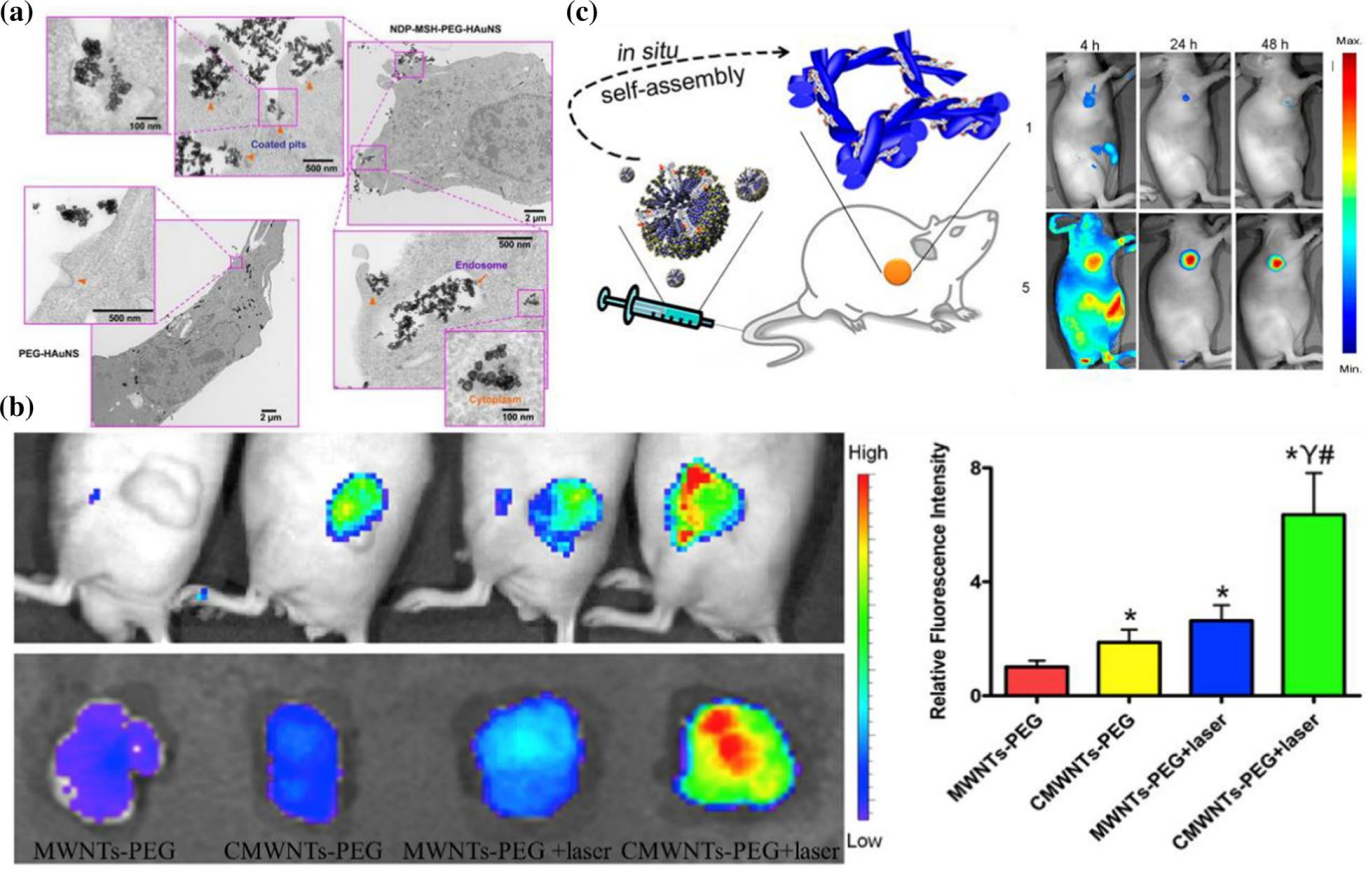
**a** TEM images of the B16/F10 cells incubated with NDP-MSH-PEG-HAuNS orPEG-HAuNS (Reprinted from Ref. [35] with permission. Copyright 2009, Chun Li). **b** In vivo self-amplified accumulation of CMWNTs-PEG in tumor tissues (Reprinted from Ref. [36] with permission. Copyright 2016, Elsevier Ltd.) **c** Scheme of in situ conversion of micelles to nanofiber after intravenous injection. Representative NIR fluorescence images of HeLa-tumor-bearing mice after intravenous administration at different times (Reprinted from Ref. [21] with permission. Copyright 2015, American Chemical Society)

Systemic administration is utilized more widely than intratumoral injection, especially for metastatic and deep tumors [30]. However, due to the phagocytosis of the reticuloendothelial system (RES) in general systemic administration, the amount of drugs that can reach the tumor site for cancer treatment is substantially less than the injection amount, and the efficacy of PTAs is hindered [31]. To achieve a high concentration of PTAs at the tumor site, nanodelivery systems have evolved from ordinary passive targeting systems to active targeting systems. As a common strategy in recent years, various targeting methods have been explored [32, 33]. Nle4-d-Phe7-α-melanocyte-stimulating hormone (NDP-MSH) is an effective receptor agonist of melanocortintype-1, which is overexpressed in many melanoma cells and combines with the melanocortintype-1 receptor with high affinity [34]. Chun Li and colleagues developed melanoma-targeted hollow gold nanospheres, which stabilized with polyethylene glycol (PEG) coating and combined with NDP-MSH (NDP-MSH-PEG-HAuNS) [35] (Fig. 2a). NDP-MSH-PEG-HAuNS and their aggregates were detected in coated pits by cell uptake experiments, and many NDP-MSH-PEG-HAuNS were detected in the cytoplasm. Moreover, the NIR laser power was 30 J/cm^2^ which is lower than the clinical data (~ 255 J/cm^2^) and can avoid unnecessary damage to surrounding normal tissues. These results indicate that NDP-MSH-PEG-HAuNS can be well phagocytized into cells to prolong the treatment time in tumors and enhance the efficacy of PTT.

Yu Hu et al. designed a self-amplified drug delivery system for tumor PTT using multiwalled carbon nanotubes (MWNTs) as a carrier and modifying CREKA (Cys-Arg-Glu-Lys-Ala) peptides with a particular affinity to fibrin as the targeting moiety (CMWNTs-PEG) [36] (Fig. 2b). This system amplified tumor targeting by a positive feedback mechanism of the coagulation response which means that fibrin is a byproduct of the coagulation reaction and can be specifically and substantially located at the site of vascular damage due to the strong signal amplification of the clotting reaction. The accumulation of CMWNTS-PEG in tumor sites was significantly higher than that of other groups, which showed an excellent tumor homing effect and realized selective killing of tumors. In addition to passive and active targeting, the exogenous magnetic field can also enhance the controlled killing of PTAs on tumors. Magnetic nanoparticles (MPSs) carrying PTAs in the blood (for example, based on superparamagnetic Fe_3_O_4_) can be redirected and accumulate in the tumor tissue under the application of an external magnetic field, selectively destroying the tumor tissue while preserving normal tissue, thus improving the selectivity and efficiency of PTT. Magnetic field-guided PTT has been successfully applied in preclinical models, truly showing its excellent clinical application prospects [37, 38].

Although active nanodelivery systems that show significant efficacy in treating tumors have been developed, most of them have been affected by unanticipated high uptake of reticuloendothelial systems (RES) (e.g., liver and spleen) [39]. An endogenous, alkaline phosphatase-triggered co-assembly strategy was proposed by Peng Huang et al. for the preparation of tumor-specific indocyanine green (ICG) nanofibers [21] (Fig. 2c). Tumor-specific supramolecular self-assemblies can be achieved through the regulation of specific enzymes. The nature of these noncovalent forces allows in situ formed nanostructures to readily incorporate drugs via the same kind of intermolecular interactions. This supramolecular system can easily avoid the undesired uptake of RES while sustaining advantages, including high tumor accumulation rate and long tumor retention time. Phosphatase-directed co-assembly processes and their diagnostic capabilities were carried out successfully at various levels, from in vitro experiments, cell experiments, and tissue simulations to in vivo experiments. The researchers observed that the tumor uptake of ICG significantly increased to 15.05 ± 3.78% ID/g after intravenous injection for 4 h, which is 25 times higher than that of free ICG (0.59 ± 0.24% ID/g). The resulting high signal-to-noise ratio (> 15) clearly distinguished the tumor from the surrounding normal tissue. Complete tumor elimination with high therapeutic accuracy was successfully achieved by laser irradiation (0.8 W/cm^2^, 5 min). In this way, this strategy successfully avoided the high uptake of RES. Nanofibers used for PTT therapy, including tumor-specific ICG-doped nanofibers, have great potential to be transformed into personalized nanomedicine treatment mediums and for clinical use in cancer treatment.

### Biomimetic targeting strategies

**Fig. 3:**
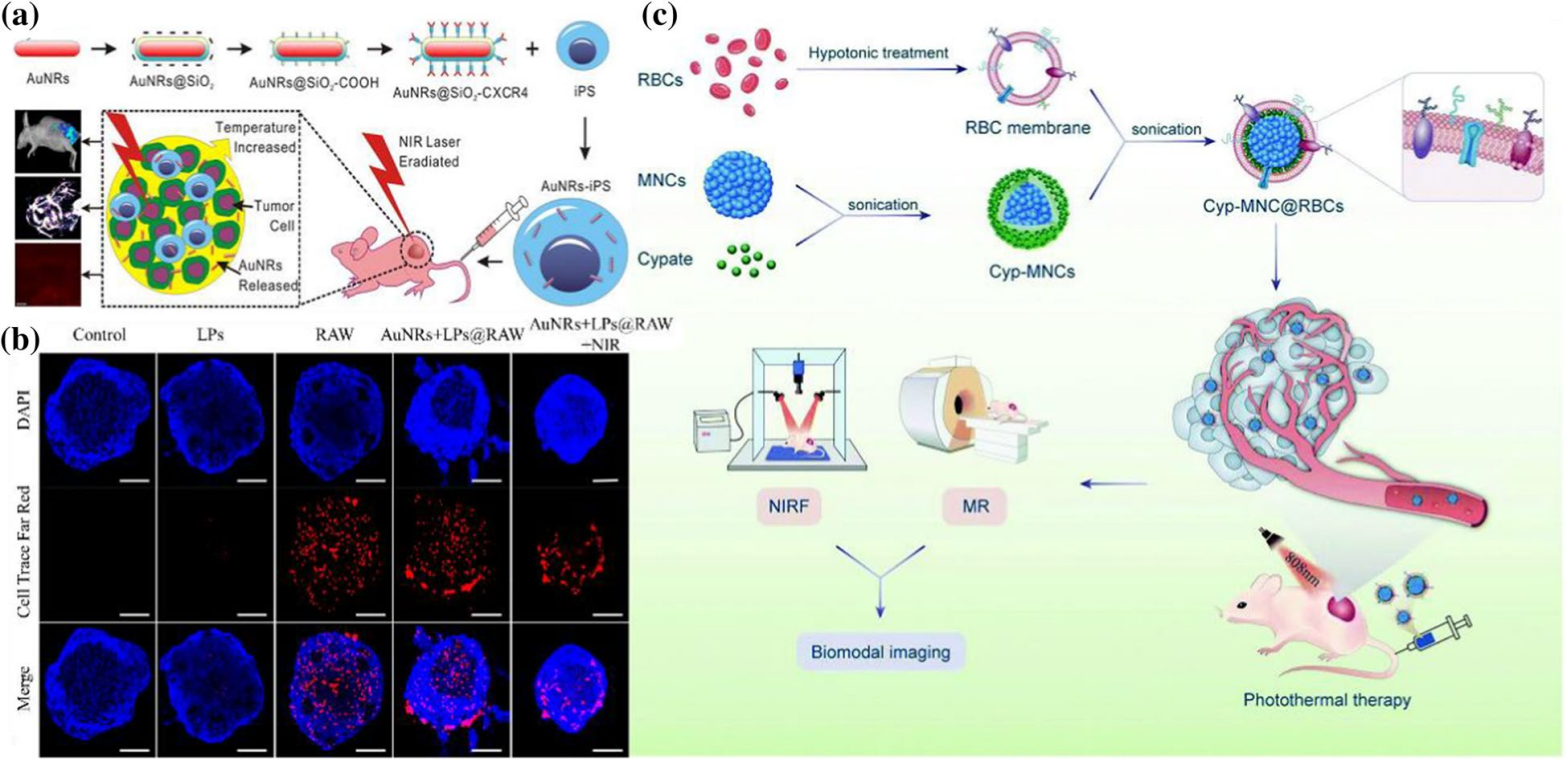
**a** AuNRs@SiO2@CXCR4 loaded human iPS cells for target delivery and intratumoral homogeneous distribution of AuNRs and enhanced photothermal therapy (Reprinted from Ref. [42] with permission. Copyright 2016, American Chemical Society.); **b** Tumor penetration of 4T1 tumorspheres by macrophages with and without nano-loading was assessed for 12 h. Compared with LPs, AUNRS + LPs@RAW showed higher penetration and tumor coverage in 4T1 cell spheres (Reprinted from Ref. [46] with permission. Copyright 2020, The Royal Society of Chemistry). **c** Schematic illustration of the preparation of Cyp-MNC@RBCs for NIR fluorescence imaging and MR imaging-guided cancer PTT (Reprinted from Ref. [18] with permission. Copyright 2018, Elsevier Ltd)

Although nanocarrier technology has made significant progress in cancer treatment research, the actual effect substantially differs from what people expect. In vivo*,* experimental data show that drugs collected into tumor cells are usually less than 5% of the injection amount. Most drugs are filtered from the body before entering tumor cells [40]. Therefore, the prolonged blood circulation time of nanoparticles in the blood is a prerequisite for targeted delivery. It is well known that specific cells can be used as bionic targeting ligands to help target or home drugs to tumors or other lesion sites, to increase blood circulation time and to improve the pharmacokinetics of drugs [41]. Some stem cells, for example, are being applied for tumor-targeted treatment. Stem cells also have a crucial role in tumor metastasis. Daxiang Cui et al. fabricated Au nanorods@SiO_2_@CXCR4 nanoparticles and loaded the prepared nanoparticles into human induced pluripotent stem cells (AuNRs-iPS) to obtain PTT nanoplatforms [42] (Fig. 3a). Due to the excellent tumor target migration capabilities of iPS cells, the researchers discovered that the Au nanorods mediated by the nanoplatform AuNRs-iPS had longer retention times and even spatial distribution. Most importantly, the discovery has demonstrated that iPS can target tumor sites and improve the efficacy of PTT to inhibit tumor growth in tumor-bearing mice.

Macrophages can realize drug homing at the tumor site through their excellent ability to target tumor migration [43, 44]. Macrophages containing therapeutic nanoparticles (including some magnetotactic bacteria) can act as Trojan horses, transporting the nanoparticles to the tumor site and destroying areas of low oxygen within the tumor to prevent malignant progression [45]. Jong-Oh Park et al. designed a macrophage-based nanotherapeutic drug delivery system to treat solid tumors by utilizing PTT, an anticancer drug, and the tumor-infiltrating properties of macrophages [46] (Fig. 3b). Compared with using nanoparticles alone, when macrophages were participating, the tumor penetration of nanoparticles was significantly improved. In addition, in vivo experiments involving local and systemic administration in tumor-bearing mice have shown that the drug delivery system of macrophage-based nanotherapeutics can effectively target and kill tumors. In addition, studies have shown that the use of red blood cell (RBC) membranes as a bionic strategy can also extend the internal blood circulation time of nanoplatforms [47]. Sheng Wang et al. prepared RBC-coated, superparamagnetic nanoclusters (MNCs); after loading with NIR cypate molecules, their NIR absorbance was dramatically improved, and efficient photothermal conversion efficiency was achieved [18] (Fig. 3c). Cyp-MNC@RBCs had a significant tumor homing ability after intravenous administration. Moreover, the tumor-bearing mice showed higher temperatures at the tumor site under laser irradiation.

In other cases, the two membranes were merged to improve the targeting capability, to increase circulation time and to reduce macrophage phagocytosis. To further enhance the therapeutic efficacy of PTT, Zhiqing Pang and colleagues fabricated an erythrocyte-cancer (RBC-M) hybrid membrane-camouflaged melanin nanoparticle (Melanin@RBC-M) platform by fusing the RBC membrane with the MCF-7 cell membrane [48]. These hybrid membrane vesicles retained both RBC cell membraned proteins and MCF-7 cell membrane proteins; the MCF-7 membrane component can significantly enhance the homotypic targeting function of Melanin@RBC-M; and the RBC membrane component can effectively reduce the cellular uptake of macrophages by Melanin@RBC-M and improve their circulation time, which greatly increases the photothermal therapeutic effect of nanomaterials.

While treating cancer with nanodelivery systems, it is often observed that most necrosis in the center of solid tumors is caused by long-term anoxicity: the availability of oxygen and glucose is insufficient for meeting the metabolic needs of malignant cells, and the destruction of the tumor in anoxic areas, especially tumor-associated macrophages (TAMs) in these areas, can effectively prevent the proliferation, growth, invasion, migration, and metastasis of malignant cells, directly affecting the mortality rate of patients [26, 47]. However, delivering therapeutic agents to the oxygen-deprived areas of the tumor is a significant challenge [25]. To solve this problem, Susan E. Clare’s team hypothesized that autonomous recruitment of tumor monocytes could be used for nanoparticle-based drug delivery and tumor therapy [44]. Because monocytes have a natural phagocytosis capacity, they can easily carry therapeutic nanoparticles to otherwise inaccessible tumor areas. After entering the tumor, the monocytes differentiate into macrophages, and then nanoparticle-laden macrophages migrate/converge to the hypoxic region of the tumor. Once in place, nanoparticle-based therapeutic functions can be activated by NIR irradiation of the tumor to destroy TAMs. Depending on the irradiation protocol, this therapeutic response can also include the destruction of adjacent tumor cells and can be combined and coordinated with other chemicals and molecular or nanoparticle-based therapies to facilitate the destruction and remission of the tumor while significantly reducing the risk of tumor regrowth and metastasis.

### Programmed targeting systems

The targeted ligand on the surface of nanoparticles can increase the affinity between nanoparticles and target cells, thus improving the uptake efficiency of cells [49]. Nevertheless, the presence of a targeted ligand can trigger an immune response, leading to the removal of nanoparticles by the mononuclear phagocyte system [50]. Most of the target ligands are hydrophobic, which can easily cause aggregation of nanoparticles in vivo. As a result, the blood circulation time of the nanoparticles is shortened [51]. However, the contradiction for aggregation between tumors and blood cannot be solved by ordinary nanoparticles. Programmed targeting strategies can confer on-demand properties on target ligands to “shield” in the bloodstream and “deshield” at tumor sites, enabling them to become suitable candidates to avoid immune system recognition and to prolong the blood circulation time [52, 53]. The use of shields or blocking groups protects the ligand from being recognized by the immune system and prolongs blood circulation. Once the shielding layer is removed at the tumor site by endogenous or exogenous stimulation, the intake of tumor cells will increase [49, 54].

**Fig. 4:**
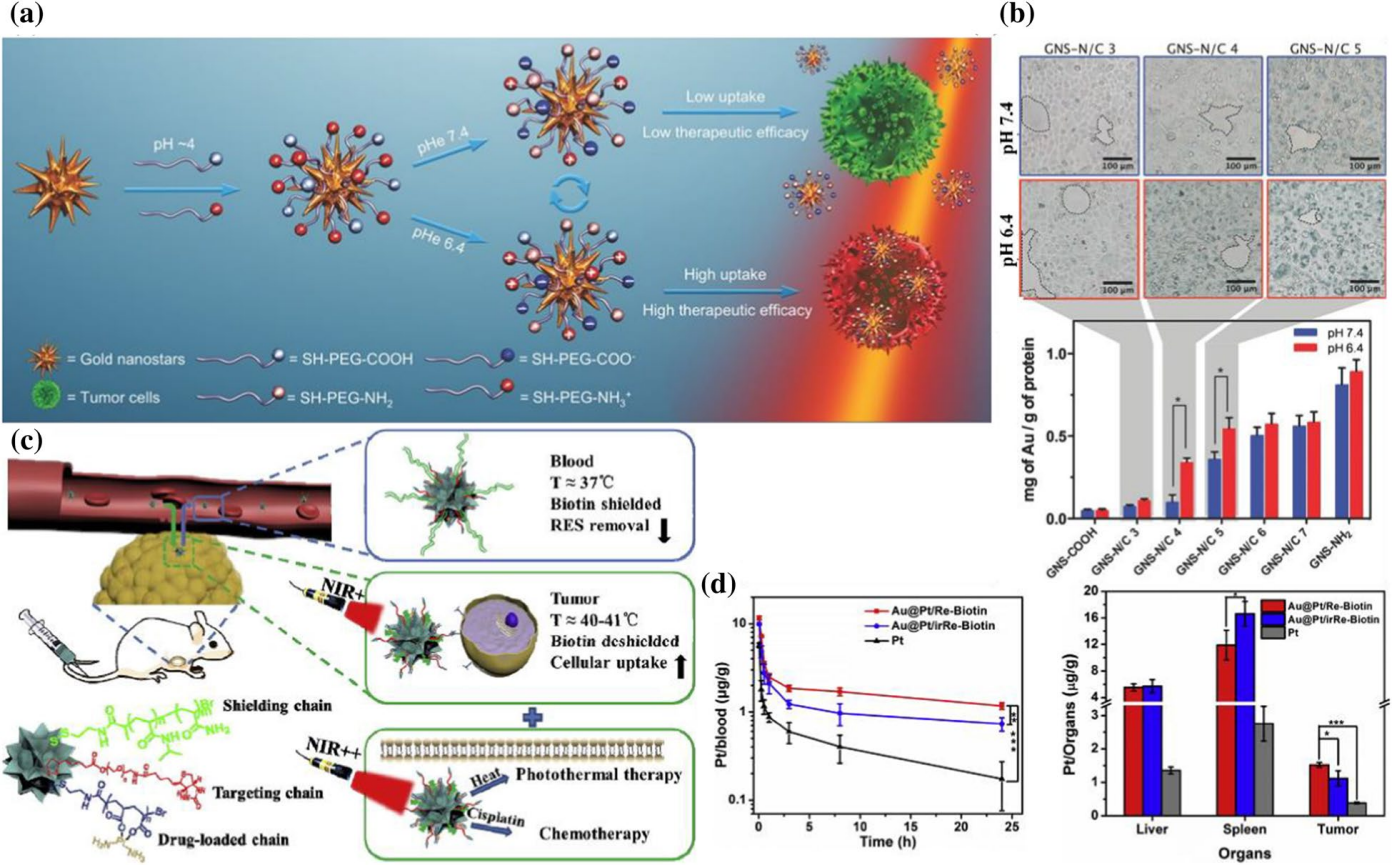
**a** Schematic illustration of the PEGylated mixed-charge GNSs and their pH-reversible cell affinity and photothermal therapeutic efficacy. **b** Optical microscopy images and cellular uptake of cells after incubation with laser for 4 h at different pH (Reprinted from Ref. [41] with permission. Copyright 2014, Wiley–VCH Verlag GmbH & Co. KGaA).** c** Schematic illustration of the based-gold nanostars temperature-responsive ligand reversible shielding system for combined photothermal therapy and chemotherapy. **d** Pharmacokinetics evaluation on Au@Pt/Re-Biotin, Au@Pt/irRe-Biotin, and cisplatin in vivo and biodistribution in the liver, spleen, and tumor (Reprinted from Ref. [49] with permission. Copyright 2018, Elsevier Ltd)

By utilizing the reversible protonation of weak electrolytic groups to pH changes, Guangming Lu et al. designed long-chain amine/carboxyl-terminated PEG decorated gold nanostars (GNSs) for PTT [41] (Fig. 4a). When incubated with HeLa cells, the degree of cellular uptake of GNS-N/C at pH 6.4 was significantly higher than that at pH 7.4 (Fig. 4b). Taking advantage of shielding nanoparticles from nonspecific interactions with normal cells/tissues before they reach tumors and after they leave tumors is crucial for the selective delivery of GNS-N/C into tumor cells, which provides a novel effective means of tumor-selective therapy. They irradiated tumors with an 808 nm laser at 1 W/cm^2^ for 5 min 24 h postinjection. Mice treated with GNS-N/C 4 (one PEGylated mixed-charge GNS with a certain surface composition) experienced an average increase of 23°C temperature, reaching an average temperature of 56°C after 5 min of treatment, which proved the excellent PTT effect of GNS-N/C 4.

Moreover, not all PTAs that arrive at the tumor would be retained in the tumor tissue. Therefore, a nanosystem that could re-shield ligands is needed to enhance the treatment selectivity of PTT. Zhi Yuan et al. researched the reversible ligand shielding strategy by a reversible ligand shielding system based on a temperature-responsive polymer [49] (Fig. 4c). The ligand biotin, cisplatin-loaded chain poly (acrylic acid)-Pt, and shielding segment thermosensitive poly(*N*-isopropylacrylamide-co-acrylamide) (P(NIPAAm-co-AAm)) were co-modified onto the surface of gold nanostars (Au@Pt/Re-Biotin). Among them, the lower critical solution temperature of P(NIPAAm-co-AAm) is approximately 39 °C, which helps to shield the ligand by the extension of P(NIPAAm-co-AAm) in the blood circulation (37°C). The ligand would be deshielded through P(NIPAAm-co-AAm) contraction utilizing the heat generated from gold nanostars upon NIR irradiation when the nanoparticles arrived at the tumor site. The results indicated that the system could extend blood circulation (1.6-fold at 24 h), reduce immune system clearance (28% lower), and enhance tumor accumulation (37% higher) effectiveness compared with the irreversible ligand shielding system (Au@Pt/irRe-Biotin) by analysis of platinum (Fig. 4d). Photothermal imaging of tumors in vivo was conducted to evaluate the photothermal conversion ability. Upon NIR (808 nm, 1 W/cm^2^, 6 min) irradiation, both Au@Pt/Re-Biotin and Au@Pt/irRe-Biotin showed apparent temperature increases, thus indicating the accumulation of gold nanostars, which possessed satisfactory photothermal conversion ability at the tumor site. In addition, this strategy showed tumor inhibition (11% higher) that was significantly superior to the irreversible system.

## Self-regulating photothermal conversion system

Selective killing of tumor cells means killing tumor cells with minimal or no damage to normal cells. Although improving the enrichment of photothermal materials in tumor sites can solve the problem to some extent, low concentrations of PTAs in normal tissue also have the ability of photothermal conversion, so how to achieve PTT without damaging normal tissue remains a challenge [55]. Therefore, is it possible to selectively increase tumor site temperature by intravenous injection with the same concentration and laser radiation in tumor and normal tissue? Assume that PTAs have a weak photothermal conversion ability in normal tissues and a robust photothermal conversion ability in tumor sites. In this case, this PTAs can selectively increase the temperature of tumor sites under inaccurate irradiation in theory, with minimal or no damage to normal cells [56]. Therefore, PTT's other strategy for selective killing is to give PTAs a self-regulating photothermal conversion capability [57, 58].

### Self-regulating of metal nanoparticles

To date, many PTAs have been discovered, including photosensitizer polymers, metal nanoparticles, carbon nanomaterials, black phosphorus (BP) based nanomaterials, and metal sulfides [59]. However, these PTAs cannot achieve a controllable photothermal conversion ability. Gold nanoparticles have become one of the most promising drug delivery materials due to their excellent biocompatibility, surface modification, and excellent photothermal conversion efficiency. The aggregation and self-assembly of spherical gold nanoparticles give them the photothermal conversion ability that they do not possess [60], making them the best candidate for materials that can regulate photothermal conversion. In the disassembled state, the NIR absorption of spherical gold nanoparticles is very weak and almost does not have a photothermal conversion capability. The absorption peak re-shifted to approximately 808 nm and generated a photothermal conversion ability in the assembled state, achieving the photothermal treatment function [61]. To meet the functional requirements of PTAs in different environments in vivo and enable them to possess unique characteristics of assembled and unassembled states, researchers have explored how to achieve responsive aggregation of small particle-sized gold nanoparticles in tumor sites.

**Fig. 5:**
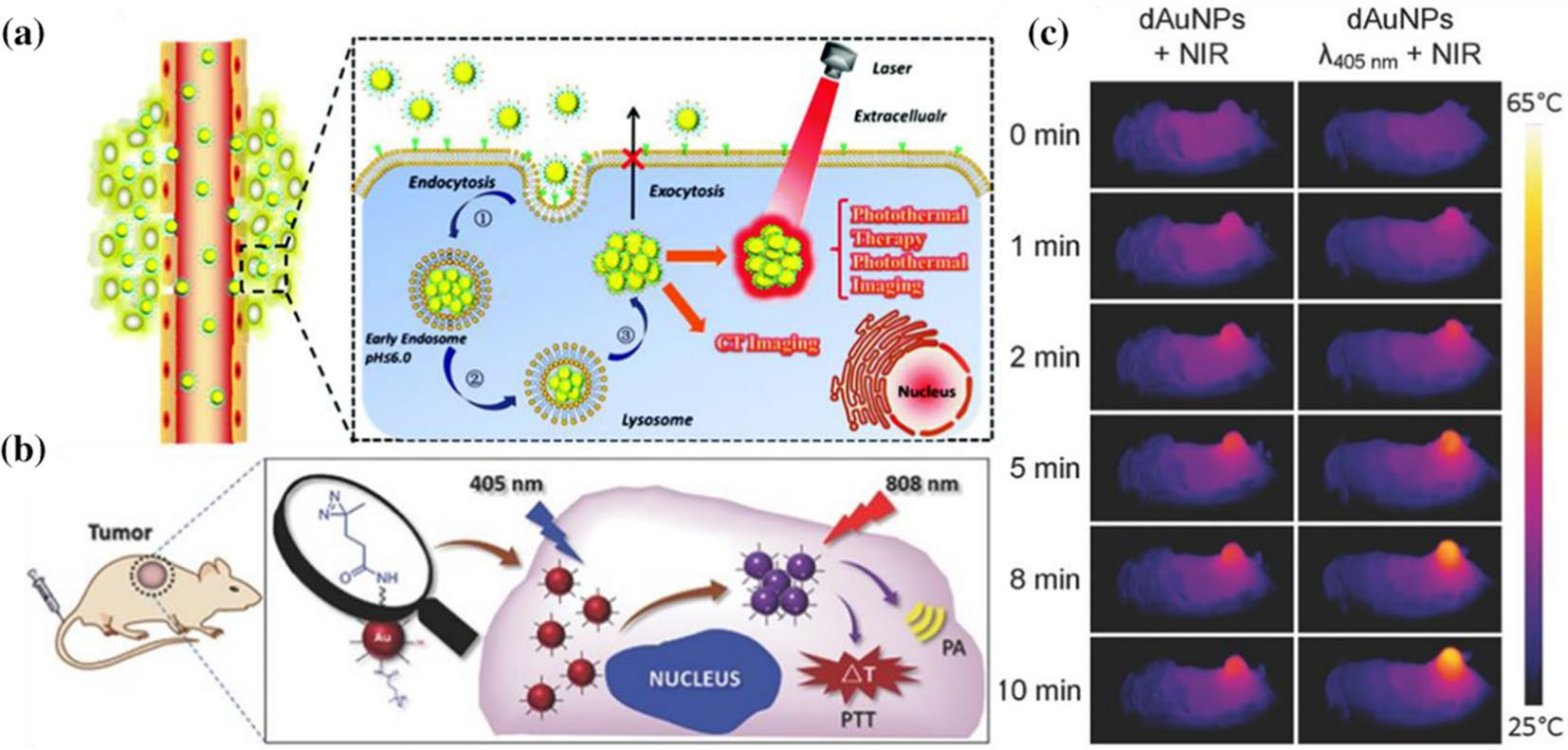
**a** Schematic image of the tumor-microenvironment-activated self-assembled Au@T SiO_2_ (Reprinted from Ref. [63] with permission. Copyright 2019, Zhi Yuan). **b** Schematic illustration of the light-triggered assembly of dAuNPs. **c** Photothermal images of tumor-bearing mice for showing the in vivo cross-linking effect of dAuNPs on tumor local temperature against irradiation time of 808 nm laser (Reprinted from Ref. [67] with permission. Copyright 2016 WILEY–VCH Verlag GmbH & Co. KGaA, Weinheim)

It has been reported that many pH-sensitive, spherical gold nanoparticles can achieve responsive self-assembly at the tumor site under the influence of the tumor microenvironment, showing strong near-infrared absorption and thermal ablation of tumors [62]. Zhi Yuan et al. [63] used a one-pot reaction to modify lipoic acid-PEG (LA-PEG), LA-PEG-Biotin, 4-mercaptobenzoic acid, and *p*-hydroxythiophenol on gold nanoparticles (Au@T), which can increase the hydrophobicity of the system under acidic conditions of pH = 6.0 to agglomerate the nanoparticles (Fig. 5a). By using dynamic light scattering to measure the size of Au@T, they discovered that after being added to pH = 6.0 PBS solution for 2 min, the size of Au@T rapidly increased from 32.8 nm (PDI = 0.229) to 249.3 nm (PDI = 0.187). Moreover, in vivo studies showed that after 8 h of exocytosis, the content of acid-responsive Au@T in HepG2 cells changed by less than 5%, while the content of non-responsive gold nanoparticles decreased by more than 10%. This finding proves that the aggregation of Au@T can prolong the residence time of nanoparticles in cells. Importantly, the temperature changes caused by different power laser irradiation were analyzed at Au@T ([Au] 150 μg/mL) in PBS buffer (pH = 6.0), and the temperature was observed to reach 50°C at 1.0 W/cm^2^ laser irradiation after 3 min. The photothermal conversion efficiency of aggregated Au@T is 30.48%, higher than the photothermal conversion efficiency of the commonly reported gold nanoparticles [64–66], which proves that aggregated Au@T has an excellent photothermal conversion ability under acidic conditions.

Haibin Shi and Mingyuan Gao developed novel light-triggered gold nanoparticles (dAuNPs) that can self-assemble in vivo by covalently cross-linking the end groups of the diazirine (DA) of the PEG_5000_ ligand on the surface of gold nanoparticles (20.5 nm) with the help of 405 nm laser irradiation [67] (Fig. 5b). After continuous irradiation for about 15 min, a second maximum absorption appeared at the shoulder is approximately 700 nm and gradually extended to the NIR of 700–900 nm. The strong surface plasmon resonance of the dAuNPs appearing in NIR renders it potentially useful for PTT. Under 808 nm laser irradiation, the cross-linked nanoparticles showed greatly enhanced photothermal effects compared with non-cross-linked nanoparticles (Fig. 5c). As a proof of concept study, the penetration depth of a 405 nm laser remains limited, but the current strategy for manipulating Au particles in vivo can be extended to other types of light-triggered assembly nanoparticles, which can be triggered by lights with wavelengths that are more suitable for clinical applications. Qiwei Tian and Shiping Yang et al. [68] proposed a novel synergistic triggering mechanism to realize the self-assembly of gold nanospheres. Au@ZIF-8 does not produce photoacoustic signal and photothermal conversion capability in normal tissue. In contrast, in the presence of overexpressed glutathione and hydrogen ions in the tumor, gold nanospheres were released from Au@ZIF-8 to form aggregates and showed solid signals for imaging and effective PTT. This work provides a new strategy for designing therapeutic agents with sequential response steps to avoid interfering with diagnostic signals from normal tissue and to reduce damage to normal tissue during treatment.

**Fig. 6:**
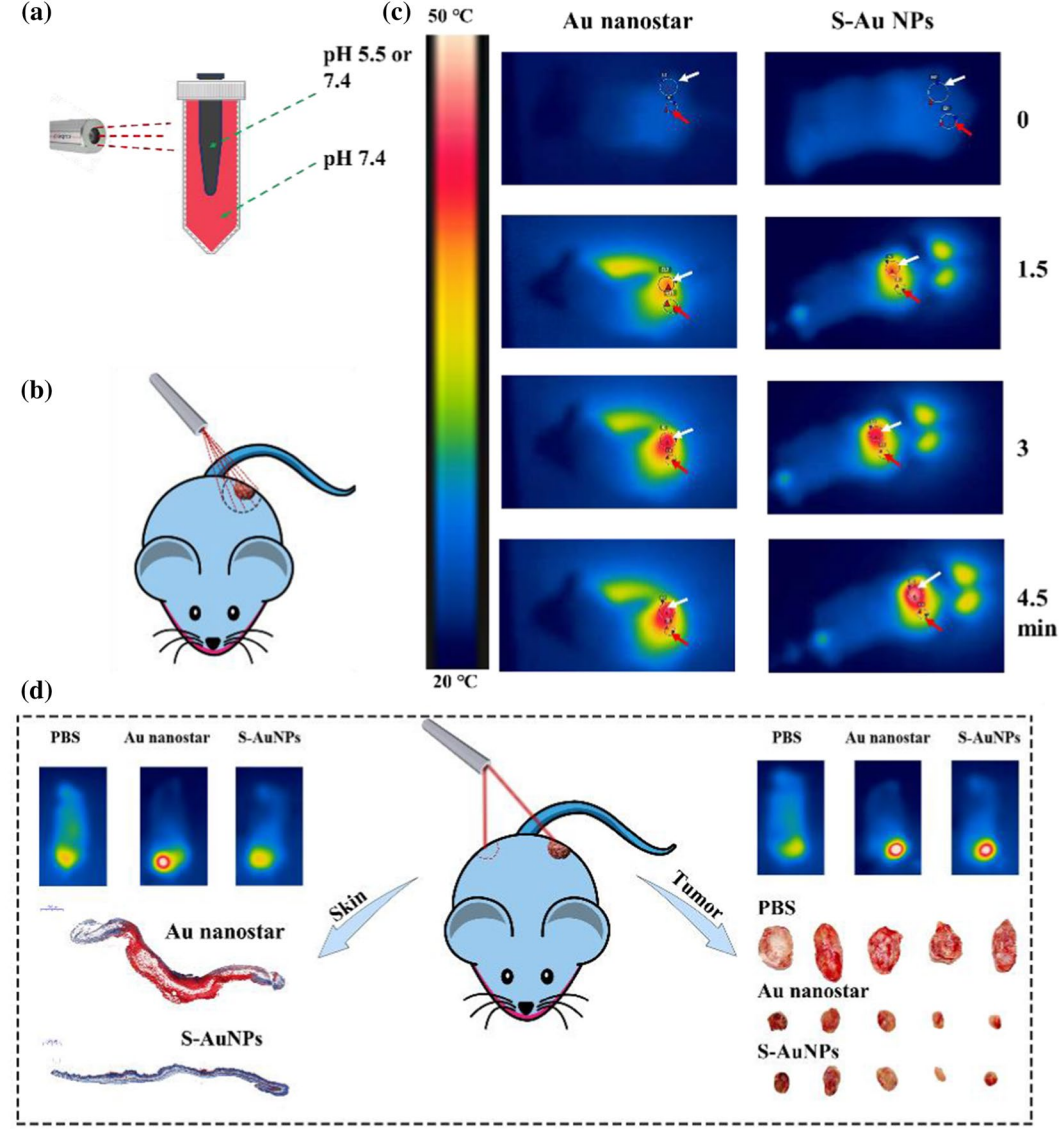
**a** Schematic diagram of the in vitro selective photothermal transformation model; **b** Schematic diagram of a “one facula” experiment; **c** Infrared thermal images and the temperature evolution of the tumor and skin tissue of mice treated with Au nanostar and S-AuNPs at 808 nm laser irradiation (3.33 W/cm^2^) at a different time; **d** Schematic illustration of specificity killing of the tumor cells under laser irradiation without skin damage (Reprinted from Ref. [61] with permission. Copyright 2022, Elsevier Ltd)

However, the excitation window of existing PTT is mainly located in the visible or NIR region, with insufficient penetration depth and relatively low interaction with tissues, limiting its thermal sensitivity effect. Therefore, Professor Zhang Dong et al. [69] developed an activatable NIR-II plasmonic theranostics system based on silica-encapsulated, self-assembled, gold nanochains (AuNCs@SiO_2_). In this study, the optical properties were precisely controlled by the structural changes of plasmonic nanoparticles in response to the tumor microenvironment, leading to accurate diagnosis and effective treatment of tumors. In normal tissue, the self-assembled gold nanochain does not change its structure and shows photoacoustic and photothermal “OFF” states in the NIR-II region. When the gold nanochain enters malignant tumor tissue, it will obtain electron conductivity through the fusion of its chain structure, and the electric field intensity is significantly enhanced, so that the surface plasmonic resonance extinction peak has a redshift, presenting an NIR-II region of photoacoustic and photothermal “ON” states. Because of the existence of “hot spots” between the gold nanoparticles and the electronic conductivity effect of the chain structure, the photoacoustic enhancement effect is significant, and the photoacoustic signal at the malignant breast tumor is greatly enhanced to realize the specific diagnosis and PTT of breast cancer. This activated strategy can realize in situ and sensitive tumor detection while effectively killing tumors, which may prominently improve the survival rate of cancer patients and introduce a new way for optical nanoengineering to become intelligent, accurate, and non-invasive in the NIR-II window. Although all these studies reported the PTT of tumors by stimuli-responsive self-assembling AuNPs, an evaluation methodology for damage to normal tissues and skin is still lacking. To validate the possibility of specifically killing tumor cells, we established an in vitro selective photothermal transformation model (Fig. 6a), a “one facula” experiment (Fig. 6b, c), and an in vivo skin-damaging assessment model (Fig. 6d) [61]. This study is the first attempt to construct an evaluation methodology for precise PTT using in vitro and in vivo models.

In addition to self-assembling gold nanoparticles, other materials can be used to self-assemble nanomaterials at tumor sites to achieve better photothermal conversion and selective thermal burn of tumors. As a rising star in the family of two-dimensional materials, BP has attracted much attention from researchers. BP has unique optical properties, and relevant reports have proven that BP two-dimensional material can serve as an efficient photothermal preparation, and phosphorus is an essential element in organisms, making its application in the biomedical field an unparalleled advantage [70–72]. Recently, Han Zhang et al. [73] conducted acid-activatable smart self-assembly of BP and polyoxometalates (POM) to produce POM@BP, which can self-assemble into large nanoparticles in the acidic tumor microenvironment, which prolongs the retention period in the tumor site, significantly improves the light absorption ability of BP nanosheets, and enhances the photothermal transformation in tumor tissue. This kind of rational design and effective customization method is promising for future scientific breakthroughs in nanomedicine.

### Self-regulating of molecule-based PTAs

To date, PTAs based on small organic molecules, such as anthocyanin dyes and porphyrins, have often been employed for PTT of tumors. Anthocyanin dyes have been shown to have excellent biophysical compatibility, improved photophysical properties, and superior near-infrared absorption, making them effective molecular PTAs, including imaging and therapy. Therefore, anthocyanin molecules with suitable photophysical ability, such as ICG, IR825, IR780, and CYPATE, have become potential candidates for PTT.

**Fig. 7:**
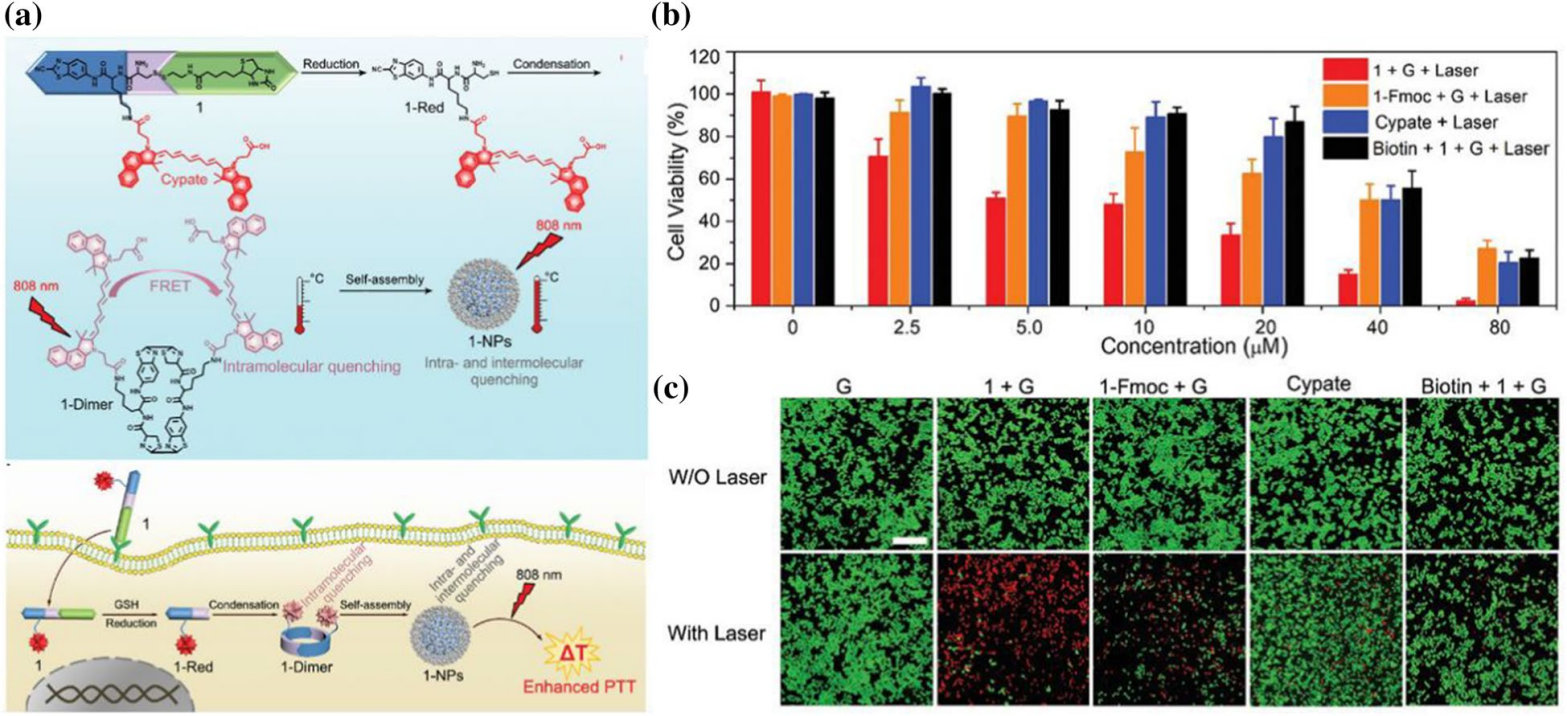
**a** Schematic illustration of reduction-controlled condensation of 1 to yield 1-Dimer, which self-assembles into 1-NPs to enhance the photothermal efficacy of the fluorophore Cypate. **b** Cell viability of HeLa cells or biotin-blocked HeLa cells after 1 h incubation with 1, 1-Fmoc, or Cypate at different concentrations, washed to remove the free compounds followed by laser irradiation and another 24 h incubation. All experiments were performed in triplicate. **c** Calcein-AM/PI live/dead staining of HeLa cells after 1 h incubation with the compounds, washed to remove the free compounds followed by another 24 h incubation, respectively (Reprinted from Ref. [13] with permission. Copyright 2019 WILEY–VCH Verlag GmbH & Co. KGaA, Weinheim)

Based on a previous project, Professor Gaolin Liang and his colleagues designed and synthesized an organic small-molecule dye, biotin-Cystamine-Cys-Lys(Cypate)-CBT, which can specifically recognize the high expression of biotin receptors in cancer cells (Fig. 7a) [13]. After being reduced by intracellular GSH, CBT-Cys forms dimers by a click condensation reaction, and the fluorescence of the two dye molecules is quenched by fluorescence resonance energy transfer (FRET). Subsequently, the dimers self-assemble in situ to form nanoparticles; intermolecular charge transfer occurs between dye molecules; and fluorescence quenching is further enhanced. Under laser irradiation, this fluorescence quenching enhanced the non-radiative excitation process of the dye, thus increasing the thermal conversion efficiency of the dye and its photothermal therapeutic effect on tumors (Fig. 7b, c). This “smart” strategy was successfully verified by enhancing the effect of PTT on living tumors. More importantly, by replacing biotin in dyes with other targeted ligands, this “smart” strategy holds promise for the PTT of other diseases.

By the same stimuli-responsive principle, Zhiyong Qian et al. prepared a nanosystem (NLG919/IR780 micelle) with the characteristics of both photothermal conversion and regulation of the tryptophan metabolism pathway to inhibit tumor growth [74]. NLG919/IR780 micelles can accumulate effectively in tumors and migrate to lymph nodes and lymphatic systems to achieve excellent tumor thermal ablation. Moreover, NLG919/IR780 inhibited the growth of the tumor margin after PTT of the primary tumor. In addition, Peng Huang et al. reported endogenous alkaline phosphatase-triggered co-assembly of indocyanine green (ICG) nanofibers [21]. In addition to increase tumor uptake, the assembled nanofibers significantly enhance the ICG NIR absorbance based on intermolecular interactions, improving the photothermal properties.

However, metallic nanoparticles and organic dyes have disadvantages, such as poor water solubility, limited tumor accumulation, and bioavailability. In addition, although the stimulus–response system has realized the self-regulating photothermal conversion ability of PTAs in vitro and animal experiments, the complexity of the in vivo environments of the body is still a major challenge for the precisely controlled photothermal conversion capability of PTAs. Therefore, researchers must conduct further studies to prove the practicality of the materials in clinical practice.

## Conclusions and perspectives

Since the concept of “precision medicine” was proposed, tumor treatment has gradually developed toward the direction of individualization and precision. The multi-subject crossing, combination, and arrival of the nanotechnology revolution have extensively promoted the application of PTT in tumor therapy [75, 76]. Due to the systemic distribution of PTAs in the body and inexact laser irradiation during treatment, PTT may cause serious damage to normal tissue. Improving the irradiation accuracy of the device and developing interventional treatment equipment can promote the development of selective PTT to a certain extent. For an ordinary NIR instrument, the selective effect of PTT depends on the difference in photothermal conversion ability between the tumor site and the normal tissue site. According to the two idea, researchers’ exploration directions can be roughly divided into two categories: (1) increasing the concentration difference between tumor sites and normal tissues and (2) endowing PTAs with self-regulating photothermal conversion capability.

### Increase the concentration difference between the tumor site and normal tissue

The photothermal conversion ability of most PTAs is in direct proportion to the concentration. Hence, increasing the concentration of photothermal nanomaterials at tumor sites is an effective method for improving the PTT accuracy. Intratumoral injection, targeting systems, biomimetic systems, and programmed targeting systems aim to increase the PTAs concentration in tumors. Although intratumoral injection can effectively cause the concentration difference between normal tissue and tumor tissue to selectively increase the temperature of the tumor site, this method cannot be directly employed for metastatic and deep tissue tumors in vivo. Additionally, many times intratumoral injections may cause to tumor metastasis. Intelligent transportation systems (targeting systems, biomimetic systems, and programmed targeting systems) enhance the uptake of tumor cells. Nevertheless, most PTAs are filtered from the body before entering tumor cells, which makes the amount of enrichment in tumor sites is far from expected. Therefore, although the method increases the concentration difference between the tumor site and normal tissue, which is feasible in theory, actual results will not materialize unless the nano-drug delivery system achieves rapid progress.

### Endowing PTAs self-regulating photothermal conversion capability

Assume that PTAs have weak photothermal conversion ability in normal tissue and strong photothermal conversion ability in the tumor site. In this case, the temperature of the tumor site will selectively increase after the same enrichment amount and infrared laser irradiation, with either minimal or no damage to normal cells. Therefore, endowing PTAs with a self-regulating photothermal conversion capability through the responsive activation of PTAs at the tumor site can achieve a better precision killing effect on tumors. Although the self-regulating photothermal conversion ability of PTAs has been realized by stimulus–response systems (such as the self-assembly of gold nanoparticles or dye molecules) in vitro and animal experiments, the complexity of the in vivo environments of the body is still a significant challenge for the precisely controlled.

### Prospects and challenges of clinical applications

As a non-invasive and spatiotemporally controllable tumor treatment method, PTT is still in the preliminary clinical research stage and has proven excellent anticancer efficacy in the laboratory and clinic [30, 77]. All the above mentioned methods are theoretically selective, and relevant experiments have not verified the safety of normal skin and body organs. For effective clinical translation of selective PTT, the following aspects should be taken into account: (1) provide in vitro and in vivo new evaluation methods to evaluate the ability of selective PTT; (2) improve the sensitivity of self-regulating PTAs to achieve selective PTT; and (3) investigate the long-term prognosis of selective PTT.

Although the complete clinical application of PTT has not fully emerged, it offers new hope for the clinical treatment of cancer. Highly selective PTT will render the construction of clinical PTT more refined and intelligent and become a new opportunity to develop clinical cancer therapy. With rational technological innovation and strategic improvements, there is a considerable scope for clinical expansion of the new PTT platform. Simultaneously, we hope that this review will provide valuable information and insights for future research into selective PTT.

## Data Availability

Not applicable.
